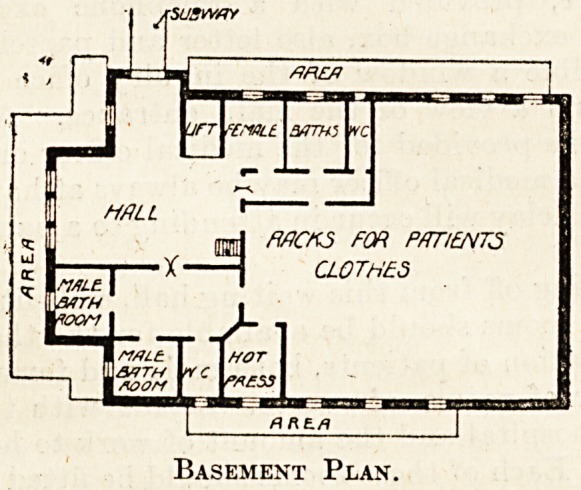# The Entrance to a General Hospital

**Published:** 1907-04-13

**Authors:** 


### HOSPITAL ADMINISTRATION.

### CONSTRUCTION AND ECONOMICS.

### THE ENTRANCE TO A GENERAL HOSPITAL.

#### I. THE GATE HOUSE OR ADMISSION BLOCK.

Its Plan and Construction.

If the efficiency of a hospital, and the regular smooth working of its departments, are to be secured, the proper management and control of the admission department is of the greatest importance. When one considers for a moment the number of applicants of all ages in various stages of disease, and the number of accident cases of every degree of severity who present themselves every day seeking admission, it will be evident that the most careful supervision must be exercised on the very threshold. It is essential that every precaution be taken against the admission of an unsuitable case, or the refusal, without careful examination, of any patient seeking admission. It is only necessary to instance the case of a patient with delirium tremens being admitted to a general ward at a late hour, or a case of infectious disease admitted through an overlook, or a case refused admission and expiring on the way home, in order to illustrate the danger and trouble which might arise should the supervision exercised over this department not be systematic, stringent, and thorough.

To secure this proper control it is necessary that the admission department should be designed on a definite plan suitable for the purposes in view. It is not sufficient to utilise any available rooms, say, in the basement of the building, where patients may be casually interviewed by a house surgeon or physician. This department should be as carefully designed and equipped as any other department of the hospital.

Within recent years much more attention has been devoted to the details of construction of this department than was formerly considered necessary, but even in the best type of hospital there is still much to be desired in this respect. It is essential for an architect in designing any building to have before him an accurate idea of all the requirements, and the uses to which each foot of space is to be put; for unless he is furnished with this information it is not possible for him to design his building so as to give effect to all the details which are so necessary. I will endeavour in a general way to enumerate the various points which an architect should have before him in designing the admission department of a general hospital.

The admission department should be conveniently placed on the ground floor of the hospital—or it may be a detached building—with a large court where ambulance wagons or other vehicles may easily pass each other on approaching or retiring from the institution. The entrance to the admission department for patients should, if possible, be entirely separate and distinct from that for the staff and students. An additional entrance should be provided for patients' friends on visiting days, in order that they may be able to enter the hospital without passing through the patients' entrance, or coming into contact with an accident case or other patient seeking admission. The main entrance door should be protected by a covered porch so that patients may be removed from the ambulance or cab to the examination room without being exposed to the weather or the gaze of inquisitive onlookers. This door should be sufficiently wide to allow two hand ambulances or barrows to pass should they require to be brought out to the ambulance or cab, and to facilitate this the floor of the entrance hall should be as nearly as possible on a level with that of the outside porch. Adjoining the entrance vestibule, lavatory accommodation should be provided for males and females who may accompany the patient. Lavatory accommodation should also be provided for porters on duty, and all lavatories should have a cut-off ventilating passage.

A recess to store ambulance barrows should adjoin the entrance, and this recess must be in proportion to the size of the hospital, in order that a hand ambulance may always be available when an accident or urgent case arrives. The vestibule should lead into a large waiting hall with an inquiry office at its entrance, provided with a telephone exchange, private exchange box, also letter and parcel racks. If possible a window of the inquiry office should command a view of the main entrance. A room should be provided for the medical officer on duty, so that a medical officer may be always at hand and that no delay will occur in attending to a patient on arrival.

Leading off from this waiting hall, well-lit examination rooms should be available for the thorough examination of patients, both male and female, the number of rooms, of course, varying with the size of the hospital and the amount of work to be overtaken. Each of these rooms should be fitted with a wash-hand basin and sink, and a plentiful supply of hot and cold water.

Two rooms, with recovery rooms adjoining, should be fitted up as small operating rooms for the treatment of minor casualties. A special room should also be furnished with an *x*-ray outfit, and arrangements should be made whereby this room can be readily darkened so that suspected fractures, etc., may be examined with the fluorescent screen.

Adjoining the admission department two small wards should be provided for the accommodation of drunks or noisy cases unfit to be placed in the general wards. To these "emergency wards" must be attached the usual bathroom and lavatory accommodation, nurses' room, ward kitchen, and urine-test room or small lavatory. These wards should have double windows in order to prevent noise being heard outside if the wards are near other buildings.

The interior walls of the admission department should, as far as possible, have a smooth and impervious surface in order that they may be easily cleaned. All angles should be avoided and all corners rounded. Although glazed tiles are open to the criticism that they have numerous joints, they probably make the most suitable wall yet devised, as they can be easily washed down at very small cost. The corridors and waiting-hall should be tiled to a height of 6 feet 6 inches, and the upper walls is not so suitable. Oak very soon becomes destroyed with water; the same trouble is experienced with pitch pine. The doors should also be made of a hard wood, preferably teak, and have no mouldings or grooves where dust can lodge. They should be wide enough to admit an ambulance barrow or bed with ease. In no case should the doors of an examination room be less than 3 feet 6 inches in width.

As an aid to a complete understanding of the varied work which has to be provided for, and the most effective method of carrying it out, I may here explain the accompanying plans of an admission block designed to embody the main principles which govern the construction of such a department.

All accidents and patients seeking admission to this hospital enter through the central gateway, and

### A Model Gate House or Admission Block for a Modern General Hospital.

Ground Floor Plan.

Basement Plan.

covered with Parian or Kean's cement, and be treated with three coats of flat paint and two coats of enamel, or, what is equally suitable and less costly, enamelette. The floors of the passages and corridors throughout the department should be covered with terrazzo, which is a mixture of Portland cement and marble chips. A margin of one foot round the rooms should be treated in the same way, and the terrazzo carried up this same distance on the wall to join the tiles. The remainder of the floors should be covered with a hard wood, such as American maple or teak. As these floors require to be frequently washed, oak on the left is shown the porters' room, where a porter is always in readiness to attend to any applicant. This room has suitable accommodation for parcels, letters, telephones, etc., and adjoining it is a small lavatory for the use of porters. At the side of the porters' room is the entrance to the central waiting-hall, which is lit from the roof. On one side of this hall are examination and dressing-rooms for males, with lavatory accommodation; and on the other side similar provision for females, with the addition of a nurses' duty room. At the end of the central hall are two operating theatres, with recovery room adjoining each; one theatre for males, and the other for females. Between these theatres are rooms for sterilisers and dressings. An *x*-ray examination-room is provided beyond the male examination-rooms on the right of the hall. In the basement, under the entrance hall and operation theatres are two bathrooms for males and two for females, with w.c.s for each. The remainder of the basement is used as a store for patients' clothes, and a hot-air chamber is provided for purposes of disinfection. The basement can be reached by a lift or by a wide staircase which is situated at the end of the waiting-hall.

(*To be continued.*)

## Figures and Tables

**Figure f1:**
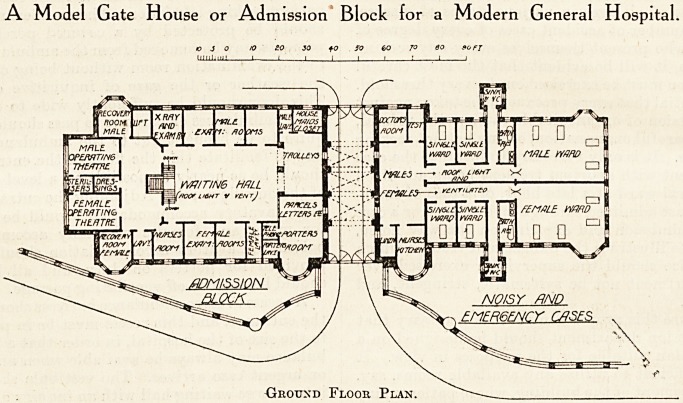


**Figure f2:**